# Tracing Penicillin Movement in Citrus Plants Using Fluorescence-Labeled Penicillin

**DOI:** 10.3390/antibiotics8040262

**Published:** 2019-12-12

**Authors:** Nabil Killiny, Pedro Gonzalez-Blanco, Yulica Santos-Ortega, Fuad Al-Rimawi, Amit Levy, Faraj Hijaz, Ute Albrecht, Ozgur Batuman

**Affiliations:** 1 Department of Plant Pathology, University of Florida, Citrus Research and Education Center, IFAS, Lake Alfred, FL 33850, USA; pcgo@ufl.edu (P.G.-B.); yulica.santosort@ufl.edu (Y.S.-O.); falrimawi@staff.alquds.edu (F.A.-R.); amitlevy@ufl.edu (A.L.); fhijaz@ufl.edu (F.H.); 2 Chemistry Department, Faculty of Science and Technology, Al-Quds University, Jerusalem 90612, Palestine; 3 Horticultural Sciences Department, University of Florida, Southwest Florida Research and Education Center, IFAS, Immokalee, FL 34142, USA; ualbrecht@ufl.edu; 4 Department of Plant Pathology, University of Florida, Southwest Florida Research and Education Center, IFAS, Immokalee, FL 34142, USA; obatuman@ufl.edu

**Keywords:** BOCILLIN™ FL-Penicillin, translocation, uptake, citrus, Huanglongbing (HLB), xylem, phloem

## Abstract

Huánglóngbìng (HLB), citrus greening, is one of the most destructive diseases of citrus plants worldwide. In North America, HLB is caused by the phloem-limited bacterium *Candidatus* Liberibacter asiaticus and is transmitted by the Asian citrus psyllid, *Diaphorina citri*. No cure exists at present, and the use of antibiotics for the control of HLB has gained interest due to the significant losses to the citrus industry. Because of unsatisfactory results when using foliar applications of antibiotics, concerns were raised regarding the uptake and translocation of these materials within trees. We, therefore, investigated a method that allows us to study the movement of antibiotic materials in citrus plants. Herein, we utilized a fluorescence-labeled penicillin, BOCILLIN™ FL-Penicillin (FL-penicillin), to study the uptake and translocation of penicillin in citrus plants. FL-penicillin was applied by puncture to the stem of young citrus seedlings and was traced by using fluorescence microscopy. After application, we detected FL-penicillin in the leaves and in the stem xylem and phloem tissues above and below the application site in both intact and partially bark-girdled citrus seedlings, indicating that it is easily taken up and transported through the plant vascular system. In addition, we detected FL-penicillin in the gut of *D. citri*, which were allowed to feed on the treated plants, suggesting translocation of this molecule into the vascular tissue. We propose that the use of fluorescent-labeled molecules could be an effective tool for understanding the uptake and translocation of antibiotics and other macromolecules in plants and insects.

## 1. Introduction

Currently, the destructive disease Huánglóngbìng (HLB), also known as citrus greening, is threatening the citrus industry worldwide. The putative phloem-limited pathogen *Candidatus* Liberibacter asiaticus, which is responsible for this disease in North America, is transmitted by *Diaphorina citri* Kuwayama, the Asian citrus psyllid. The *D. citri* transmits the *Ca*. L. asiaticus while feeding on the phloem sap of young citrus leaves [1]. Since the discovery of HLB in China in 1919 [2], several control methods have been attempted, including the application of insecticides to control *D. citri*, removal of infected trees, thermotherapy, and enhanced nutritional programs [3]. Although control of *D. citri* using insecticides is considered to be the most effective method for the management of HLB, this method does not cure *Ca*. L. asiaticus-infected trees or stop the spread of the disease entirely. The development of tolerant citrus cultivars is one of the most effective solutions for living with HLB; unfortunately, most commercial citrus cultivars are highly susceptible to the disease [4].

The use of antibiotics was first suggested for the control of HLB disease in the 1970s after HLB was found to be caused by a bacterium [5]. Early studies showed that direct injection of tetracycline or penicillin into the trunk of *Ca*. L. asiaticus-infected trees could significantly reduce HLB symptoms [6,7,8,9,10]. Due to the significant economic losses since the arrival of HLB to North America, the use of antibiotics for the control of HLB disease has regained interest in the citrus industry. Recent reports showed that penicillin and streptomycin can reduce *Ca*. L. asiaticus titers and rescue HLB-infected plants [11,12]. Other bactericides, such as ampicillin, carbenicillin, penicillin, cefalexin, rifampicin, and sulfadimethoxine, were also found to be effective against *Ca*. L. asiaticus [10,12,13].

The efficacy of antibiotics in vitro does not guarantee an equivalent efficacy in planta [14]. Despite positive reports on the efficacy of antibiotics against *Ca*. L. asiaticus, widespread foliar applications of antibiotics in the field has had limited success. This raised concerns about the uptake and movement of these materials into and throughout the plant and prompted the investigation of trunk injection as a more efficient application method. The efficacy of using trunk applications of oxytetracycline hydrochloride, penicillin, and streptomycin on reducing *Ca*. L. asiaticus titer was recently documented [15], but detailed information on the movement of these antibiotics within the plant is scarce.

The efficiency of agrochemicals, such as insecticidal and herbicidal compounds, depends on their ability to reach their site of action. For example, herbicides should reach the meristem to kill the weed, whereas insecticides should move via the xylem and/or the phloem in order to kill sucking and chewing insects [16]. The xylem is a part of the long-distance transport system in plants in which water, nutrients, and other compounds are carried upward and are distributed throughout the plant. This upward transport occurs because of the negative hydrostatic pressure that is created through evaporation of water on the leaf surface (transpiration). The phloem is responsible for the long-distance movement of sugars and other organic compounds throughout plants. Translocation in the phloem is bidirectional and occurs from source tissue, such as photosynthesizing leaves to sink tissues such as roots [16].

Because the efficacy of antibiotics mainly depends on their uptake, distribution, and persistence in the plant, a detailed understanding of antibiotic movement is necessary to evaluate their efficacy for controlling HLB disease. This knowledge may allow us to (1) better formulate a set of recommendations that could improve citrus tree health, (2) minimize the amount of antibiotics applied in the field, and (3) reduce the impact of antibiotics on the environment. Therefore, the objective of this study was to evaluate a fluorescence-labeled penicillin to trace its uptake and movement throughout the plant. The fluorescent molecule BOCILLIN™ FL-Penicillin (FL-penicillin) was utilized as a tool to visualize the translocation of a complex molecule from the site of application through the vascular system of young citrus plants and finally into the digestive system of the insect vector.

## 2. Results

### 2.1. Translocation of FL-Penicillin in Intact Plants

**Figure 1:**
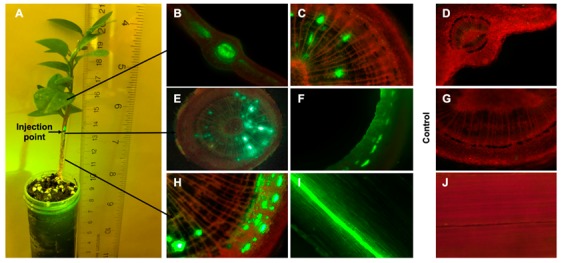
Distribution of BOCILLIN™ FL-Penicillin in Mexican lime seedlings 18 h after stem application. (**A**) Mexican lime seedling showing application site and tissue types that were investigated by fluorescence microscopy; (**B**) leaf blade at 2 cm above the application site; (**C**) leaf petiole at 2 cm above the application site; (**D**) leaf blade of the control plant; (**E**) stem at the application site; (**F**) removed bark phloem tissue; (**G**) stem of the control plant; (**H**) stem at 2 cm below the application site; (**I**) inner view of the bark 2 cm below the application site; and (**J**) inner view of the bark from the control plant.

The distribution of FL-penicillin in the different tissues of citrus plants after stem application is shown in Figure 1A. Fluorescent micrographs revealed that the compound was taken up through the punctures and translocated to distant parts of the plant as fluorescence was detected in the leaf blade (Figure 1B) and petiole (Figure 1C) 2 cm above the application site. In the area above the application site, we found FL-penicillin in the inner woody part of the stem containing the xylem (Figure 1E), and in the bark surrounding the xylem containing the phloem (Figure 1F). Furthermore, fluorescence was observed in the stem xylem and phloem 2 cm below the application site (Figure 1H,I). In general, the fluorescence intensity above the application site exceeded that below the application site, indicating a predominant upward movement of penicillin. We did not detect fluorescence in any of the tissues of the water-treated control plants (Figure 1D,G,J). The higher fluorescence levels in the xylem than in the phloem demonstrate that the xylem was the main transport route for the FL-penicillin.

### 2.2. Translocation of FL-Penicillin in Girdled Plants

**Figure 2:**
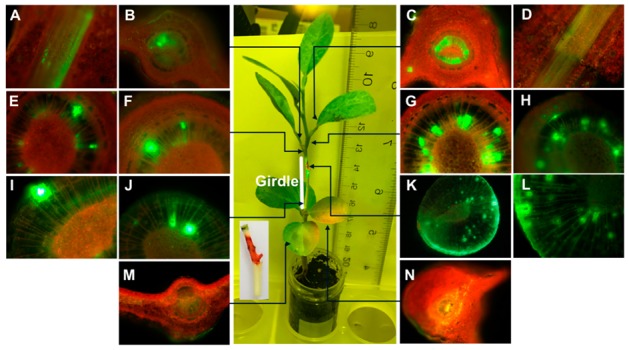
Distribution of fluorescent BOCILLIN™ FL-Penicillin in girdled Mexican lime seedling after stem application. The center picture shows the application site in the girdled area and the different locations that were investigated by fluorescence microscopy. (**A**) Inner view of the bark at 1.5 cm; (**B**) leaf blade at 1.5 cm above the application site; (**C**) leaf blade at 2 cm above the application site; (**D**) inner view of the bark at 2 cm above the application point; (**E**) magnified view of the stem 0.5 cm above the application site; (**F**) stem 0.5 cm above the application site; (**G**) stem 1 cm above the application site; (**H**) magnified view of the stem 1 cm above the application site; (**I**) magnified view of the stem (girdled) 2 cm below the application site; (**J**) stem (girdled) 2 cm below application site; (**K**) application site (girdled); (**L**) magnified view of the application site; (**M**) leaf blade at 4 cm below the application site; and (**N**) leaf blade at 3 cm below the application site.

In order to gain a better insight to the roles xylem and phloem may play in the translocation of FL-penicillin in citrus, we first girdled the stems of the Mexican lime plants 5 cm above the soil level and then applied FL-penicillin through this girdled area on the stem. Microscopic analysis of the tissues dissected from the partially bark-girdled citrus seedlings showed that FL-penicillin was taken up and translocated to distant parts of the plant, as detectable florescence was present in the leaves, xylem, and phloem of the stem above the application site (the girdled area) (Figure 2A–H). The FL-penicillin was detected in the xylem (Figure 2I–J) at about 2 cm below the application point (Figure 2K,L). Fluorescence was more intense in the tissues above (Figure 2A–H) than below the girdled area (Figure 2I,J,M,N). The fluorescence intensity in the xylem tissues was higher than in the phloem tissues (Figure 2A–H). The presence of FL-penicillin mainly in the xylem vessels above the girdled area demonstrates that the translocation occurred mainly upward in the xylem.

### 2.3. Tracing of Penicillin in D. citri

**Figure 3:**
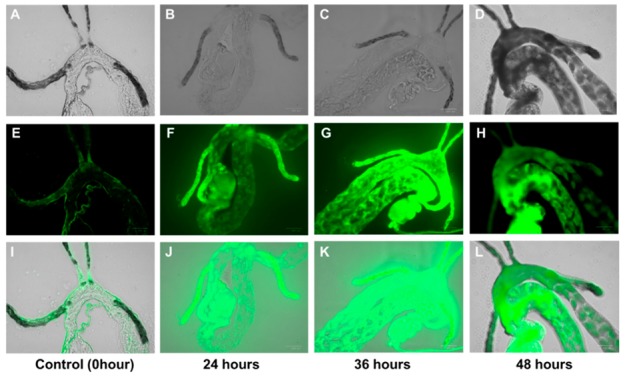
*D. citri* guts under transmitted light and fluorescence microscopy after 0, 24, 36, and 48 h feeding on Mexican lime seedlings, incubated in fluorescent penicillin solution. (**A**–**D**) Images with light microscope; (**E**–**H**) images with fluorescence microscope; and (**I**–**L**) overlaid images of light microscope and fluorescence microscope. Note the increasing gradient in the fluorescence due to the accumulation of penicillin over the feeding period, with a corresponding gradient of midgut cell death (darkened cells in 48 h photos).

To further assess the translocation of the FL-penicillin in citrus plants, we used an additional yet indirect method in which adult *D. citri* insects were allowed to feed on young leaves of Mexican lime seedlings treated with FL-penicillin. The FL-penicillin was detected in the digestive system (i.e., foregut, midgut, and hindgut) in *D. citri* 24 h after feeding on FL-penicillin-treated plants (Figure 3). Penicillin initially accumulated in the filter chamber (Figure 3B,F,J) and then moved to other parts of the gut (Figure 3C,D,G,H,K,L). We observed malformation and death in the gut cells of *D. citri* 36 h and 48 h after feeding on penicillin-treated plants (Figure 3C,D,G,H,K,L). The presence of FL-penicillin in the insect gut demonstrated that *D. citri* consumed the compound from the plant vascular system.

## 3. Discussion

The efficiency of the antimicrobial compounds in planta highly depends on their translocation and ability to reach their site of action. For example, antimicrobial compounds designed to target phloem- or xylem-limited bacteria should be able to reach the phloem or xylem. Uptake of foliar- or trunk-applied agrochemicals can be affected by many factors, including temperature, humidity, soil moisture, and physiological activity of the tree [17]. In addition, the uptake of compounds that are applied by trunk injection is affected by the xylem anatomy, including vessel arrangement and lumen size [18]. Ring-porous trees, such as citrus trees, are able to absorb fluids quickly, whereas diffuse-porous and non-porous trees have a moderate or low absorption rate, respectively [18].

In this work, we investigated the uptake and translocation of penicillin in citrus plants using fluorescent penicillin. FL-penicillin is the most common fluorophore antibiotic conjugate in which a boron-dipyrromethene (BODIPY) is linked to the phenyl side chain of the β-lactam [19]. FL-penicillin was developed in 1999, and it has been successfully used to study the mode of action of β-lactam antibiotics and detect the penicillin-binding proteins (PBPs) in *Escherichia coli*, *Pseudomonas aeruginosa*, and *Streptococcus pneumonia* [19,20]. In fact, the use of this FL-penicillin offers a sensitive, rapid, and safe (nonradioactive) detection of PBPs [20]. We demonstrated that FL-penicillin is an ideal tool to trace uptake, movement, and translocation of penicillin in plants and insects. Our results demonstrate that the use of fluorescent penicillin is easier, safer, and faster than the use of radioactive or phosphor imaging, which is often used to visualize insecticide and herbicide movement in plants [16].

Our results showed evidence of FL-penicillin uptake and translocation throughout the girdled and non-girdled citrus plants. Fluorescence was detected in the woody part of the stem, as well as in the surrounding bark, suggesting its presence not only in the xylem but also in the phloem of the treated plants. In accordance with the upward movement of water in the xylem, more fluorescence was detected above than below the application site. In fact, downward movement to the root could be limited by capillary action and root pressure.

The high level of fluorescence in the xylem of intact and girdled citrus seedlings indicated that the xylem was the main route of penicillin translocation. The detection of fluorescence in the bark above the application site suggests a possible radial translocation of penicillin from the xylem to the phloem. The bidirectional exchange of substances other than water between the xylem and phloem was reported for sulfate, phosphate, sodium, potassium, chloride, calcium, magnesium, amino acids, and amino compounds [21]. We therefore hypothesize that relatively large-sized macromolecules such as FL-penicillin may also be translocated from the xylem to the phloem.

The detection of fluorescence in the gut of *D. citri* after feeding on FL-penicillin-treated plants suggests that the compound was present in the vascular tissue of these plants. Sap-sucking insects such as *D. citri*, ingest from the phloem and xylem sap of plants [22]. Interestingly, in addition to fluorescence, we observed malformation of the guts of *D. citri* that consumed the FL-penicillin. This malformation could have resulted from the bactericidal effect of penicillin on the endosymbionts of *D. citri*, which helps insects digest food and provides essential nutrients [23]. A recent report also demonstrated that trunk injections of penicillin could affect the bacterial community structure in the root of citrus trees [24].

## 4. Materials and Methods

### 4.1. Plant Materials

Healthy Mexican lime (*Citrus aurantifolia*) seedlings were used in this study. Seeds were purchased from Lyn Citrus Seed, Inc. (Arvin, CA, USA) and individually planted in plastic cones (20 × 4 cm) containing Sungro professional growing mix (Sungro Horticulture, Agawam, MA, USA). Germinated seedlings were kept in a greenhouse (28 °C ± 1 °C, 60% ± 5% relative humidity, 16:8 h light/dark photoperiod) at the Citrus Research and Education Center (CREC), University of Florida, Lake Alfred, Florida. Seedlings were watered twice weekly. All the seedlings were about three months old and about 15 ± 5 cm tall.

### 4.2. Insect Culture

Colonies of *D. citri* were reared on HLB-free curry leaf plants (*Bergera* (*Murraya*) *koenigii*) in a growth room maintained at 28 ± 1 °C and 60% ± 5% relative humidity, and a 16:8 (light/dark) photoperiod. Only adult psyllids were collected for feeding experiments.

### 4.3. Application of Labeled Penicillin

**Figure 4:**
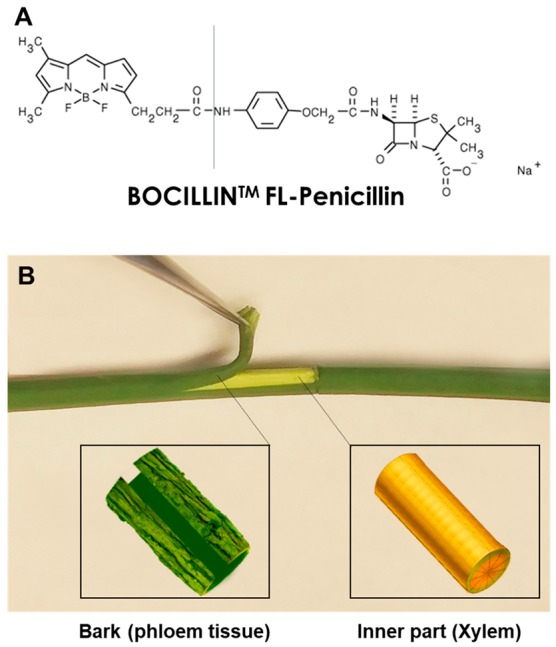
Materials used in this study. (**A**) Structure of BOCILLIN™ FL-Penicillin; (**B**) dissection of the stem bark into bark tissue (representing the phloem) and wood (representing the xylem).

BOCILLIN™ FL-Penicillin sodium salt (Figure 4A) was purchased from Thermo Fisher Scientific (Waltham, MA, USA). The compound was applied through several punctures that were made on the stem at 7 cm above the soil level using a 0.3 mm diameter needle. A 5 µL aliquot of BOCILLIN™ FL-Penicillin (500 ppm) was applied at the punctured area using a pipettor. The application of fluorescent penicillin was repeated three more times in 20 min intervals. Five seedlings were used for experimentation. The seedlings were kept for 18 h at ambient temperature and were then dissected into root, stem bark, stem wood, and leaves and analyzed using a fluorescent microscope as described below. The purpose of this experiment was to simulate the application of antibiotics through trunk injection and monitor movement and translocation throughout the plants. This experiment was repeated three times.

Five additional seedlings were girdled by completely removing a 4 cm-long strip of bark from the entire circumference of the stem at 5 cm above the soil level. Several punctures were made in the middle of the girdled area, and FL penicillin was applied as described above. The seedlings were kept for 18 h at ambient temperature. At the end of the incubation time, plants were dissected into root, stem bark, stem wood, and leaves and tissues were investigated using a fluorescent microscope. The purpose of this experiment was to determine whether translocation of FL-penicillin can occur from the xylem to the phloem of a plant. Hereafter, bark and wood tissue extracted from the stems tissue will be referred to as “phloem” and “xylem” (Figure 4B).

### 4.4. Feeding of D. citri on Penicillin-Treated Citrus Cuttings

The Mexican lime seedling was cut into two parts at about 10 cm above the ground, and the leafy upper part (5 cm) was incubated in 50 µL of FL-penicillin inside a 0.5 mL centrifuge tube. The centrifuge tube was covered with parafilm, and the cutting was placed in a 15 mL plastic centrifuge tube with 20 adult *D. citri*. Five *D. citri* were collected after 24, 36, and 48 h and examined by stereoscope. This experiment was repeated three times. Control psyllids were kept on citrus cuttings incubated in water.

### 4.5. Microscopy

The plants were dissected into the different parts as described above and were manually sectioned into 0.5 mm-thin segments for fluorescent microscopy using sterile, double-edged stainless-steel blades (Personnna, Los Angeles, CA, USA). All the samples were covered with a drop of mineral oil to avoid any autofluorescence.

For microscopic observations, a Carl Zeiss AxioScope A1 fluorescent microscope (Carl Zeiss Microscopy GmbH, Göttingen, Germany) equipped with a Zeiss Axio Cam ICc1, with filter Set 43 or Rhodamine filter from Zeiss (Ex: BP 545/25, Em: BP 525/50) for red and green fluorescence, was used.

Low-magnification images were captured with a modified WildHeerbrugg stereoscope (Wild Heerbrugg Instruments, Ltd., Heerbrugg, Switzerland) with white light. For fluorescent images, a NIGHTSEA™ Stereo Microscope Fluorescence Adapter with light and filter set (royal blue with green-only bandpass filter, SFA-LFS-GO) was added (Nightsea, Lexington, MA, USA). Images were captured with a Canon PowerShotS3 IS (Martin Microscope Co., Easley, SC). Images of untreated control tissue and FL-penicillin-treated tissue were taken under identical conditions.

For observation of the *D. citri* digestive system after feeding on plants treated with FL-penicillin, insects were decapitated with a razor blade, and the guts were dissected and mounted on glass slides with a drop of 10 mM HEPES (4-(2-hydroxyethyl)-1-piperazineethanesulfonic acid)/10 mM sucrose solution. Fluorescence was visualized using a Bio-Rad ZOE Fluorescent Cell Imager (Bio-Rad Laboratories, Hercules, CA, USA). Images of the insect guts were captured with brightfield light and the green channel fluorescence light source. Images from both channels were overlaid to obtain two-color micrographs.

## 5. Conclusions

In conclusion, our results demonstrate that BOCILLIN™ FL-Penicillin can be successfully used to study the translocation of penicillin in citrus plants and *D. citri* tissues. Our results show that the movement of the FL-penicillin occurred primarily in the xylem vessels with the transpiration stream. The presence of fluorescence in the bark suggested that penicillin moved radially from the xylem to the phloem during its transport through the plant. In addition, the presence of FL-penicillin in the gut of *D. citri* suggested translocation of this molecule into the vascular tissue. Our results indicate that fluorescent-labelled antibiotics could be a valuable tool for studying the movement of antibiotics in plants and insects.
